# Supplementary material for: Joint Modeling of Longitudinal Biomarker and Survival Outcomes with the Presence of Competing Risk in Nested Case-Control Studies with Application to the TEDDY Microbiome Dataset
Source: bioRxiv. 2025 May 28:2025.05.23.655653. Preprint. [Version 1] doi: 10.1101/2025.05.23.655653 (PMC12154706; doi:10.1101/2025.05.23.655653)
Supplement: Supplement 1 [file media-1.pdf]

Table S1: Performance of all methods for point and 95% confidence interval estimation of β_1 and β_2 under **Scenario 2** ($\beta_1 = 0.1$ and $\beta_2 = 0.1$).

		β_1						β_2					
**m**[1]	**Method**	**Bias**	**SE**[2]	**ESE**[3]	**MSE**[4]	**CI-L**[5]	**ECP**[6]	**Bias**	**SE**	**ESE**	**MSE**	**CI-L**	**ECP**
1	Oracle	0.002	0.072	0.073	0.005	0.284	0.943	0.000	0.044	0.044	0.002	0.172	0.953
	fJM-NCC	0.003	0.074	0.077	0.006	0.292	0.940	0.001	0.046	0.048	0.002	0.182	0.939
	wJM-NCC	0.004	0.082	0.082	0.007	0.320	0.953	0.002	0.058	0.058	0.003	0.227	0.957
	wJM-NCC(Fisher)	0.004	0.073	0.082	0.007	0.284	0.911	0.002	0.044	0.058	0.003	0.172	0.864
	JM	-0.026	0.072	0.083	0.008	0.282	0.933	-0.030	0.044	0.045	0.003	0.171	0.899
	CLR	-0.002	0.098	0.101	0.010	0.383	0.951	-0.009	0.058	0.059	0.004	0.228	0.947
3	Oracle	-0.003	0.072	0.073	0.005	0.284	0.946	-0.003	0.044	0.043	0.002	0.172	0.961
	fJM-NCC	-0.002	0.074	0.074	0.005	0.288	0.946	-0.002	0.045	0.044	0.002	0.178	0.959
	wJM-NCC	-0.003	0.075	0.075	0.006	0.294	0.943	-0.002	0.049	0.049	0.002	0.191	0.946
	wJM-NCC(Fisher)	-0.003	0.072	0.075	0.006	0.284	0.939	-0.002	0.044	0.049	0.002	0.172	0.920
	JM	-0.019	0.072	0.070	0.005	0.282	0.952	-0.016	0.044	0.043	0.002	0.171	0.937
	CLR	-0.007	0.078	0.079	0.006	0.305	0.945	-0.011	0.047	0.046	0.002	0.184	0.951
5	Oracle	0.003	0.073	0.073	0.005	0.284	0.952	0.001	0.044	0.044	0.002	0.172	0.948
	fJM-NCC	0.002	0.073	0.074	0.005	0.288	0.952	0.001	0.045	0.046	0.002	0.176	0.940
	wJM-NCC	0.003	0.074	0.074	0.006	0.290	0.948	0.001	0.047	0.047	0.002	0.182	0.952
	wJM-NCC(Fisher)	0.003	0.072	0.074	0.006	0.284	0.945	0.001	0.044	0.047	0.002	0.172	0.930
	JM	-0.007	0.072	0.073	0.005	0.282	0.949	-0.008	0.044	0.044	0.002	0.171	0.940
	CLR	-0.004	0.073	0.075	0.006	0.288	0.947	-0.008	0.045	0.045	0.002	0.174	0.944

1. Control-to-case ratio, i.e., the number of controls per case in the NCC sub-cohort
2. Estimated standard error
3. Empirical standard error
4. Mean squared error
5. Average length of the 95% confidence intervals
6. Empirical coverage probability of the 95% confidence interval

Table S2: Performance of all methods for point and 95% confidence interval estimation of β_1 and β_2 under **Scenario 2** ($\beta_1 = 0.2$ and $\beta_2 = 0.1$).

		β_1						β_2					
**m**[1]	**Method**	**Bias**	**SE**[2]	**ESE**[3]	**MSE**[4]	**CI-L**[5]	**ECP**[6]	**Bias**	**SE**	**ESE**	**MSE**	**CI-L**	**ECP**
1	Oracle	-0.003	0.071	0.069	0.005	0.279	0.963	0.001	0.044	0.044	0.002	0.173	0.941
	fJM-NCC	0.000	0.074	0.072	0.005	0.289	0.967	0.002	0.047	0.048	0.002	0.183	0.944
	wJM-NCC	-0.002	0.082	0.080	0.006	0.320	0.957	0.001	0.058	0.059	0.003	0.227	0.947
	wJM-NCC(Fisher)	-0.002	0.071	0.080	0.006	0.280	0.927	0.001	0.044	0.059	0.003	0.173	0.866
	JM	-0.044	0.071	0.070	0.007	0.277	0.903	-0.038	0.044	0.044	0.003	0.172	0.866
	CLR	-0.019	0.098	0.098	0.010	0.385	0.953	-0.006	0.059	0.059	0.004	0.230	0.945
3	Oracle	0.001	0.072	0.071	0.005	0.281	0.952	0.001	0.044	0.044	0.002	0.173	0.947
	fJM-NCC	0.003	0.073	0.073	0.005	0.286	0.946	0.002	0.046	0.045	0.002	0.179	0.956
	wJM-NCC	0.001	0.075	0.075	0.006	0.294	0.956	0.001	0.049	0.049	0.002	0.192	0.947
	wJM-NCC(Fisher)	0.001	0.072	0.075	0.006	0.281	0.944	0.001	0.044	0.049	0.002	0.173	0.915
	JM	-0.019	0.071	0.071	0.005	0.278	0.952	-0.018	0.044	0.044	0.002	0.172	0.928
	CLR	-0.016	0.078	0.077	0.006	0.305	0.952	-0.007	0.047	0.048	0.002	0.185	0.946
5	Oracle	0.001	0.072	0.077	0.006	0.281	0.934	0.003	0.044	0.046	0.002	0.173	0.941
	fJM-NCC	0.002	0.073	0.077	0.006	0.285	0.938	0.003	0.045	0.047	0.002	0.177	0.939
	wJM-NCC	0.001	0.073	0.078	0.006	0.287	0.945	0.003	0.047	0.049	0.002	0.183	0.942
	wJM-NCC(Fisher)	0.001	0.072	0.078	0.006	0.280	0.932	0.003	0.044	0.049	0.002	0.173	0.928
	JM	-0.013	0.071	0.075	0.006	0.278	0.930	-0.009	0.044	0.045	0.002	0.171	0.934
	CLR	-0.017	0.073	0.079	0.006	0.287	0.923	-0.005	0.045	0.046	0.002	0.176	0.942

1. Control-to-case ratio, i.e., the number of controls per case in the NCC sub-cohort
2. Estimated standard error
3. Empirical standard error
4. Mean squared error
5. Average length of the 95% confidence intervals
6. Empirical coverage probability of the 95% confidence interval

Table S3: Performance of all methods for point estimation of additional parameters under **Scenario 1**($\beta_1 = \beta_2 = 0.$). Parameters include the fixed slope γ, standard deviation (log) of the random intercept θ, standard deviation (log) of random error σ, and the fixed effect α.

		$\gamma = 0.1$			$\log(\theta) = \log\sqrt{2}$			$\log(\sigma) = 0$			$\alpha = -0.2$		
**m[1]**	**Method**	**Bias**	**SE[2]**	**ESE[3]**	**Bias**	**SE**	**ESE**	**Bias**	**SE**	**ESE**	**Bias**	**SE**	**ESE**
1	Oracle	0.000	0.033	0.033	-0.005	0.008	0.009	0.001	0.004	0.004	-0.004	0.101	0.100
	fJM-NCC	0.003	0.111	0.109	-0.008	0.027	0.029	0.001	0.013	0.013	-0.004	0.101	0.100
	wJM-NCC	0.005	0.144	0.138	-0.007	0.038	0.039	0.000	0.017	0.017	-0.004	0.141	0.103
	wJM-NCC(Fisher)	0.005	0.033	0.138	-0.007	0.008	0.039	0.000	0.004	0.017	-0.004	0.101	0.103
	JM	0.004	0.120	0.115	0.007	/	0.031	0.024	/	0.012	0.194	0.101	0.026
3	Oracle	0.000	0.033	0.032	-0.005	0.008	0.009	0.001	0.004	0.004	-0.001	0.101	0.103
	fJM-NCC	-0.002	0.078	0.082	-0.005	0.020	0.020	0.001	0.009	0.009	-0.001	0.101	0.103
	wJM-NCC	-0.004	0.086	0.086	-0.005	0.022	0.022	0.001	0.010	0.010	0.000	0.115	0.105
	wJM-NCC(Fisher)	-0.004	0.033	0.086	-0.005	0.008	0.022	0.001	0.004	0.010	0.000	0.101	0.105
	JM	-0.002	0.086	0.088	0.009	/	0.021	0.025	/	0.009	0.184	0.101	0.019
5	Oracle	0.000	0.033	0.033	-0.005	0.008	0.009	0.001	0.004	0.004	0.000	0.101	0.100
	fJM-NCC	-0.001	0.064	0.067	-0.006	0.016	0.017	0.001	0.008	0.008	0.000	0.101	0.100
	wJM-NCC	0.001	0.068	0.070	-0.006	0.018	0.018	0.001	0.008	0.008	0.000	0.109	0.101
	wJM-NCC(Fisher)	0.001	0.033	0.070	-0.006	0.008	0.018	0.001	0.004	0.008	0.000	0.101	0.101
	JM	-0.001	0.071	0.069	0.009	/	0.018	0.025	/	0.007	0.173	0.101	0.020

1. Control-to-case ratio, i.e., the number of controls per case in the NCC sub-cohort
2. Estimated standard error
3. Empirical standard error

Table S4: Performance of all methods for point estimation of additional parameters under **Scenario 2**($\beta_1 = 0$ and $\beta_2 = 0.1$). Parameters include the fixed slope γ, standard deviation (log) of the random intercept θ, standard deviation (log) of random error σ, and the fixed effect α.

		$\gamma = 0.1$			$\log(\theta) = \log\sqrt{2}$			$\log(\sigma) = 0$			$\alpha = -0.2$		
**m[1]**	**Method**	**Bias**	**SE[2]**	**ESE[3]**	**Bias**	**SE**	**ESE**	**Bias**	**SE**	**ESE**	**Bias**	**SE**	**ESE**
1	Oracle	-0.001	0.033	0.034	-0.005	0.008	0.009	0.001	0.004	0.004	-0.002	0.101	0.098
	fJM-NCC	-0.002	0.111	0.113	-0.007	0.027	0.028	0.000	0.013	0.013	-0.002	0.101	0.098
	wJM-NCC	-0.002	0.144	0.136	-0.008	0.038	0.039	0.001	0.017	0.018	-0.002	0.141	0.100
	wJM-NCC(Fisher)	-0.002	0.033	0.136	-0.008	0.008	0.039	0.001	0.004	0.018	-0.002	0.101	0.100
	JM	0.001	0.120	0.122	0.010	/	0.029	0.024	/	0.013	0.194	0.101	0.027
3	Oracle	0.001	0.033	0.032	-0.005	0.008	0.009	0.001	0.004	0.004	-0.002	0.101	0.101
	fJM-NCC	-0.002	0.078	0.077	-0.007	0.020	0.020	0.001	0.009	0.009	-0.002	0.101	0.101
	wJM-NCC	-0.005	0.086	0.084	-0.007	0.022	0.022	0.001	0.010	0.010	-0.002	0.115	0.102
	wJM-NCC(Fisher)	-0.005	0.033	0.084	-0.007	0.008	0.022	0.001	0.004	0.010	-0.002	0.101	0.102
	JM	-0.005	0.086	0.082	0.009	/	0.022	0.025	/	0.009	0.182	0.101	0.019
5	Oracle	0.001	0.033	0.033	-0.005	0.008	0.009	0.001	0.004	0.004	0.002	0.101	0.097
	fJM-NCC	0.000	0.064	0.063	-0.006	0.016	0.017	0.001	0.008	0.008	0.002	0.101	0.097
	wJM-NCC	0.000	0.068	0.067	-0.005	0.018	0.017	0.001	0.008	0.008	0.002	0.109	0.098
	wJM-NCC(Fisher)	0.000	0.033	0.067	-0.005	0.008	0.017	0.001	0.004	0.008	0.002	0.101	0.098
	JM	0.000	0.071	0.068	0.010	/	0.018	0.025	/	0.007	0.173	0.101	0.021

1. Control-to-case ratio, i.e., the number of controls per case in the NCC sub-cohort
2. Estimated standard error
3. Empirical standard error

Table S5: Performance of all methods for point estimation of additional parameters under **Scenario 2**($\beta_1 = 0.1$ and $\beta_2 = 0.1$). Parameters include the fixed slope γ, standard deviation (log) of the random intercept θ, standard deviation (log) of random error σ, and the fixed effect α.

		$\gamma = 0.1$			$\log(\theta) = \log\sqrt{2}$			$\log(\sigma) = 0$			$\alpha = -0.2$		
**m**[1]	**Method**	**Bias**	**SE**[2]	**ESE**[3]	**Bias**	**SE**	**ESE**	**Bias**	**SE**	**ESE**	**Bias**	**SE**	**ESE**
1	Oracle	0.001	0.033	0.034	-0.004	0.008	0.009	0.001	0.004	0.004	0.004	0.101	0.102
	fJM-NCC	0.001	0.111	0.112	-0.006	0.027	0.029	0.000	0.013	0.013	0.004	0.101	0.102
	wJM-NCC	0.001	0.144	0.141	-0.005	0.038	0.038	0.001	0.017	0.018	0.005	0.142	0.105
	wJM-NCC(Fisher)	0.001	0.033	0.141	-0.005	0.008	0.038	0.001	0.004	0.018	0.005	0.101	0.105
	JM	0.004	0.120	0.120	0.011	/	0.033	0.024	/	0.013	0.193	0.101	0.042
3	Oracle	-0.001	0.033	0.032	-0.005	0.008	0.009	0.001	0.004	0.004	0.001	0.101	0.101
	fJM-NCC	-0.001	0.078	0.080	-0.005	0.020	0.020	0.002	0.009	0.010	0.001	0.101	0.101
	wJM-NCC	-0.004	0.086	0.088	-0.005	0.022	0.022	0.002	0.010	0.011	0.001	0.115	0.102
	wJM-NCC(Fisher)	-0.004	0.033	0.088	-0.005	0.008	0.022	0.002	0.004	0.011	0.001	0.101	0.102
	JM	-0.003	0.086	0.086	0.011	/	0.022	0.026	/	0.009	0.182	0.101	0.020
5	Oracle	0.000	0.033	0.032	-0.005	0.008	0.009	0.001	0.004	0.004	0.000	0.101	0.104
	fJM-NCC	0.004	0.064	0.067	-0.005	0.016	0.017	0.001	0.008	0.008	0.000	0.101	0.104
	wJM-NCC	0.003	0.068	0.070	-0.004	0.018	0.018	0.001	0.008	0.008	0.000	0.109	0.106
	wJM-NCC(Fisher)	0.003	0.033	0.070	-0.004	0.008	0.018	0.001	0.004	0.008	0.000	0.101	0.106
	JM	0.004	0.071	0.071	0.011	/	0.018	0.026	/	0.007	0.173	0.101	0.022

1. Control-to-case ratio, i.e., the number of controls per case in the NCC sub-cohort
2. Estimated standard error
3. Empirical standard error

Table S6: Performance of all methods for point estimation of additional parameters under **Scenario 2**($\beta_1 = 0.2$ and $\beta_2 = 0.1$). Parameters include the fixed slope γ, standard deviation (log) of the random intercept θ, standard deviation (log) of random error σ, and the fixed effect α.

		$\gamma = 0.1$			$\log(\theta) = \log\sqrt{2}$			$\log(\sigma) = 0$			$\alpha = -0.2$		
**m[1]**	**Method**	**Bias**	**SE[2]**	**ESE[3]**	**Bias**	**SE**	**ESE**	**Bias**	**SE**	**ESE**	**Bias**	**SE**	**ESE**
1	Oracle	0.001	0.033	0.032	-0.005	0.008	0.009	0.001	0.004	0.004	-0.004	0.101	0.098
	fJM-NCC	-0.001	0.110	0.112	-0.006	0.027	0.028	0.001	0.013	0.013	-0.005	0.101	0.098
	wJM-NCC	-0.003	0.144	0.145	-0.004	0.038	0.039	0.000	0.017	0.018	-0.006	0.142	0.102
	wJM-NCC(Fisher)	-0.003	0.033	0.145	-0.004	0.008	0.039	0.000	0.004	0.018	-0.006	0.101	0.102
	JM	-0.001	0.121	0.119	0.014	/	0.031	0.025	/	0.012	0.190	0.101	0.028
3	Oracle	-0.001	0.033	0.033	-0.005	0.008	0.009	0.001	0.004	0.004	0.001	0.101	0.100
	fJM-NCC	0.003	0.077	0.078	-0.006	0.020	0.020	0.001	0.009	0.009	0.001	0.101	0.100
	wJM-NCC	0.001	0.085	0.086	-0.006	0.022	0.023	0.001	0.010	0.010	0.002	0.115	0.102
	wJM-NCC(Fisher)	0.001	0.033	0.086	-0.006	0.008	0.023	0.001	0.004	0.010	0.002	0.101	0.102
	JM	0.002	0.086	0.084	0.012	/	0.021	0.025	/	0.009	0.183	0.101	0.021
5	Oracle	-0.001	0.033	0.034	-0.005	0.008	0.009	0.001	0.004	0.004	0.000	0.101	0.100
	fJM-NCC	0.000	0.064	0.065	-0.005	0.016	0.016	0.001	0.008	0.007	0.000	0.101	0.100
	wJM-NCC	0.000	0.068	0.068	-0.004	0.018	0.017	0.001	0.008	0.008	0.000	0.109	0.102
	wJM-NCC(Fisher)	0.000	0.033	0.068	-0.004	0.008	0.017	0.001	0.004	0.008	0.000	0.101	0.102
	JM	0.000	0.071	0.071	0.012	/	0.018	0.026	/	0.007	0.173	0.101	0.022

1. Control-to-case ratio, i.e., the number of controls per case in the NCC sub-cohort
2. Estimated standard error
3. Empirical standard error

Table S7: Performance of all methods for point estimation of additional parameters under **Scenario 2**($\beta_1 = 0.3$ and $\beta_2 = 0.1$). Parameters include the fixed slope γ, standard deviation (log) of the random intercept θ, standard deviation (log) of random error σ, and the fixed effect α.

		$\gamma = 0.1$			$\log(\theta) = \log\sqrt{2}$			$\log(\sigma) = 0$			$\alpha = -0.2$		
**m[1]**	**Method**	**Bias**	**SE[2]**	**ESE[3]**	**Bias**	**SE**	**ESE**	**Bias**	**SE**	**ESE**	**Bias**	**SE**	**ESE**
1	Oracle	0.000	0.033	0.033	-0.004	0.008	0.008	0.001	0.004	0.004	-0.004	0.101	0.097
	fJM-NCC	0.007	0.108	0.104	-0.006	0.027	0.027	0.000	0.013	0.012	-0.004	0.101	0.097
	wJM-NCC	0.004	0.144	0.137	-0.005	0.038	0.038	0.000	0.017	0.016	-0.003	0.143	0.101
	wJM-NCC(Fisher)	0.004	0.033	0.137	-0.005	0.008	0.038	0.000	0.004	0.016	-0.003	0.101	0.101
	JM	0.008	0.121	0.113	0.019	/	0.029	0.025	/	0.012	0.191	0.101	0.089
3	Oracle	0.001	0.033	0.031	-0.004	0.008	0.009	0.001	0.004	0.004	0.000	0.101	0.100
	fJM-NCC	0.001	0.077	0.076	-0.005	0.020	0.020	0.001	0.009	0.009	0.000	0.101	0.100
	wJM-NCC	0.002	0.085	0.084	-0.005	0.022	0.022	0.001	0.010	0.010	0.000	0.116	0.102
	wJM-NCC(Fisher)	0.002	0.033	0.084	-0.005	0.008	0.022	0.001	0.004	0.010	0.000	0.101	0.102
	JM	0.000	0.086	0.083	0.016	/	0.021	0.026	/	0.009	0.182	0.101	0.023
5	Oracle	0.000	0.033	0.033	-0.005	0.008	0.009	0.001	0.004	0.004	0.001	0.101	0.102
	fJM-NCC	0.002	0.064	0.064	-0.005	0.016	0.016	0.000	0.008	0.008	0.001	0.101	0.102
	wJM-NCC	0.000	0.068	0.068	-0.005	0.018	0.017	0.000	0.008	0.008	0.002	0.109	0.106
	wJM-NCC(Fisher)	0.000	0.033	0.068	-0.005	0.008	0.017	0.000	0.004	0.008	0.002	0.101	0.106
	JM	0.002	0.071	0.070	0.014	/	0.018	0.025	/	0.007	0.174	0.101	0.024

1. Control-to-case ratio, i.e., the number of controls per case in the NCC sub-cohort
2. Estimated standard error
3. Empirical standard error

Table S8: Estimation results and hypothesis testing p-values for the species that associated with appearance of IAA-first (β_1) and GADA-first (β_2) identified by various methods.

Species	fJM-NCC	wJM-NCC	CLR
Megasphaera elsdenii	(-40.50, -48.40)[1]<br>(< 0.001, < 0.001)[2]	(-38.90, -46.40)<br>(1, 1)	(8.97, 15.64)<br>(1, 1)
Bifidobacterium dentium	(4.75, 4.68)<br>(< 0.001, < 0.001)	(-5.98, -5.05)<br>(0.199, 1.00)	(-10.66, -2.26)<br>(0.639, 1)
Lactobacillus delbrueckii	(-80.20, 22.70)<br>(0.001, 1)	(-58.80, 42.20)<br>(0.322, 1)	(67.25, -11.26)<br>(1, 1)
Bacteroides heparinolyticus	(-35.80, 5.20)<br>(0.001, 1)	(-35.70, 5.25)<br>(0.040, 1)	(11.06, 14.79)<br>(1, 1)
Ethanoligenens harbinense	(-43.3, 26.7)<br>(0.002, 1)	(-30.9, 30.7)<br>(0.138, 1)	(105.518, 87.925)<br>(0.153, 1)
Acidaminococcus intestini	(-36.7, -16)<br>(0.002, 1)	(-36.1, -15.2)<br>(1, 1)	(22.051, -5.407)<br>(1, 1)
Bacteroides sp. A1C1	(-7, 0.412)<br>(0.003, 1)	(-5.59, 1.1)<br>(0.082, 1)	(-1.161, -3.188)<br>(1, 1)
Escherichia marmotae	(116, -52.6)<br>(0.004, 1)	(116, -52.8)<br>(0.118, 1)	(23.832, -8.44)<br>(1, 1)
Oscillibacter valericigenes	(-31.9, 24.4)<br>(0.012, 1)	(-26.4, 24.6)<br>(0.060, 1)	(78.761, 46.646)<br>(0.401, 1)
Corynebacterium argentoratense	(-50.8, -2.79)<br>(0.013, 1)	(-50.5, -2.53)<br>(0.225, 1)	(10.472, -15.795)<br>(1, 1)
Gordonibacter urolithinfaciens	(-34.8, 10.7)<br>(0.013, 1)	(-34.7, 10.6)<br>(0.050, 1)	(4.748, -4.489)<br>(1, 1)
Ruminococcus albus	(-31.6, 23.6)<br>(0.014, 1)	(-23.2, 27.2)<br>(0.543, 1)	(98.702, 69.7)<br>(0.028, 1)
Ruminococcaceae bacterium CPB6	(-34, 27.8)<br>(0.014, 1)	(-26.2, 29.6)<br>(0.242, 1)	(107.033, 79.485)<br>(0.024, 1)
Caproiciproducens sp. NJN-50	(-35, 30.4)<br>(0.014, 1)	(-28.9, 31.3)<br>(0.091, 1)	(91.71, 89.276)<br>(0.138, 1)
Paraprevotella xylaniphila	(-29.7, 17.6)<br>(0.015, 1)	(-28.7, 17.9)<br>(0.116, 1)	(14.925, 10.281)<br>(1, 1)
Bacillus cereus	(-101, -86.2)<br>(0.019, 1)	(-100, -86.1)<br>(0.048, 1)	(-1.47, 136.413)<br>(1, 1)
Bacteroides intestinalis	(-18.4, 0.944)<br>(0.024, 1)	(-10.9, 6)<br>(1, 1)	(10.539, 7.884)<br>(1, 1)
Escherichia albertii	(77.2, -41.4)<br>(0.026, 1)	(56.4, -54.7)<br>(1, 1)	(-0.466, -78.615)<br>(1, 1)
Alistipes finegoldii	(-7.84, 4.15)<br>(0.029, 1)	(-7.29, 4.35)<br>(0.061, 1)	(2.133, 0.458)<br>(1, 1)
Ruminococcus bicirculans	(-6.59, 6.16)<br>(0.033, 1)	(-5.63, 7.24)<br>(0.281, 1)	(14.039, 4.357)<br>(0.729, 1)
Christensenella sp. Marseille-P3954	(-32.3, 23.9)<br>(0.033, 1)	(-32, 23.9)<br>(0.012, 1)	(88.909, 62.8)<br>(0.168, 1)

Streptococcus thermophilus	(-16.8, 2.91)<br>(0.033, 1)	(-16.5, 2.77)<br>(0.088, 1)	(0.081, -3.191)<br>(1, 1)
Corynebacterium variabile	(-314, -362)<br>(0.033, 0.315)	(-314, -362)<br>(0.003, 0.375)	(57.346, -97.577)<br>(1, 1)
Ruminococcus champanellensis	(-23.3, 22.1)<br>(0.038, 1)	(-23.1, 22.2)<br>(0.027, 1)	(77.238, 34.679)<br>(0.040, 1)
Alistipes sp. 5CBH24	(-29, 26.6)<br>(0.043, 1)	(-29, 26.4)<br>(0.008, 1)	(19.585, 7.32)<br>(1, 1)
Flintibacter sp. KGMB00164	(-18.7, 13.8)<br>(0.045, 1)	(-15.9, 13.9)<br>(0.156, 1)	(50.723, 18.737)<br>(0.282, 1)
Bifidobacterium	(-1.3, 0.059)<br>(0.059, 1)	(0.491, 1.76)<br>(1, 1)	(2.064, -0.768)<br>(1, 1)
Clostridiales bacterium CCNA10	(-10.2, 7.09)<br>(0.067, 1)	(-8.69, 7.02)<br>(0.132, 1)	(31.273, 17.425)<br>(0.087, 1)
Ruminococcus sp. JE7A12	(-11.1, 9.24)<br>(0.073, 1)	(-8.77, 11.7)<br>(1, 1)	(25.473, 20.154)<br>(0.591, 1)
Veillonella parvula	(6.63, -5.88)<br>(0.075, 1)	(6.08, -6.16)<br>(0.354, 1)	(-3.582, -4.292)<br>(1, 1)
Bifidobacterium breve	(1.35, 0.454)<br>(0.075, 1)	(0.968, 0.131)<br>(1, 1)	(-0.84, 0.552)<br>(1, 1)
Bacteroides dorei	(-3.41, -1.62)<br>(0.075, 1)	(-2.04, -0.081)<br>(1, 1)	(-0.878, 1.489)<br>(1, 1)
Intestinimonas butyriciproducens	(-25.9, 21)<br>(0.084, 1)	(-25.6, 21.2)<br>(0.083, 1)	(93.448, 37.092)<br>(0.039, 1)
Adlercreutzia equolifaciens	(-18.5, 11.8)<br>(0.090, 1)	(-18.4, 11.8)<br>(0.208, 1)	(31.881, 10.543)<br>(1, 1)
Paeniclostridium sordellii	(-43.9, 19.9)<br>(0.093, 1)	(-21.9, 28.6)<br>(1, 1)	(40.45, 70.657)<br>(1, 1)
Roseburia hominis	(-7.71, 6)<br>(0.095, 1)	(-5.89, 6.82)<br>(1, 1)	(16.917, 18.309)<br>(0.596, 1)
Collinsella aerofaciens	(2.85, 7.19)<br>(0.276, <0.001)	(-0.603, 3.86)<br>(1, 0.789)	(0.395, 5.9)<br>(1, 1)
Lactobacillus paracasei	(-11.4, -22.3)<br>(0.242, 0.002)	(3.26, -24.7)<br>(1, 1)	(-7.275, -19.401)<br>(1, 1)
Alistipes sp. 5CPEGH6	(-23.8, 20)<br>(0.170, 1)	(-23.6, 19.9)<br>(0.098, 1)	(50.942, 3.761)<br>(1, 1)
[Eubacterium] sulci	(-29.8, 12.2)<br>(1, 1)	(-39.7, 61.1)<br>(1, 1)	(167.44, 122.065)<br>(0.009, 1)
Lachnospiraceae bacterium GAM79	(-6.4, 5.56)<br>(0.134, 1)	(-4.76, 6.91)<br>(1, 1)	(19.413, 10.612)<br>(0.020, 1)
Turicibacter sp. H121	(-15.9, 14.2)<br>(1, 1)	(-10.4, 17.2)<br>(1, 1)	(48.578, 32.014)<br>(0.048, 1)
Anaerobutyricum hallii	(-5.14, 4.31)<br>(0.566, 1)	(-3.79, 5.02)<br>(1, 1)	(17.125, 13.296)<br>(0.062, 1)
Campylobacter jejuni	(-13.4, 0.894)<br>(1, 1)	(-13.4, 1.1)<br>(1, 1)	(90.038, -28.752)<br>(0.062, 1)

[1] The point estimates and [2] the hypothesis testing p-values of (β_1, β_2) for the null hypothesis $H_0: \beta_1 = 0$ and $H_0: \beta_2 = 0$ respectively.
